# Body height as risk factor for emphysema in COPD

**DOI:** 10.1038/srep36896

**Published:** 2016-11-22

**Authors:** Massimo Miniati, Matteo Bottai, Ivana Pavlickova, Simonetta Monti

**Affiliations:** 1 Department of Experimental and Clinical Medicine, University of Florence, 50134 Florence, Italy; 2 Unit of Biostatistics, Department of Environmental Medicine, Karolinska Institutet, 17177 Stockholm, Sweden; 3 Institute of Clinical Physiology, National Research Council of Italy, 56124 Pisa, Italy; 4 “Gabriele Monasterio” Tuscany Foundation, 56124 Pisa, Italy

## Abstract

Pulmonary emphysema is a phenotypic component of chronic obstructive pulmonary disease (COPD) which carries substantial morbidity and mortality. We explored the association between emphysema and body height in 726 patients with COPD using computed tomography as the reference diagnostic standard for emphysema. We applied univariate analysis to look for differences between patients with emphysema and those without, and multivariate logistic regression to identify significant predictors of the risk of emphysema. As covariates we included age, sex, body height, body mass index, pack-years of smoking, and forced expiratory volume in one second (FEV_1_) as percent predicted. The overall prevalence of emphysema was 52%. Emphysemic patients were significantly taller and thinner than non-emphysemic ones, and featured significantly higher pack-years of smoking and lower FEV_1_ (P < 0.001). The prevalence of emphysema rose linearly by 10-cm increase in body height (r^2^ = 0.96). In multivariate analysis, the odds of emphysema increased by 5% (95% confidence interval, 3 to 7%) along with one-centimeter increase in body height, and remained unchanged after adjusting for all the potential confounders considered (P < 0.001). The odds of emphysema were not statistically different between males and females. In conclusion, body height is a strong, independent risk factor for emphysema in COPD.

Pulmonary emphysema is currently defined as “a condition of the lung characterized by abnormal, permanent enlargement of the airspaces distal to the terminal bronchiole accompanied by destruction of their walls, without obvious fibrosis”^1^,^2^. Emphysema is a phenotypic component of chronic obstructive pulmonary disease (COPD), bearing substantial lung function impairment and poor prognosis^3^. Emphysemic patients feature lower body mass index, less exercise tolerance, and worse quality of life than non-emphysemic ones^4^,^5^,^6^,^7^.

Also, emphysema is a strong predictor of reduced survival^8^, independently of coexisting cardiovascular or metabolic disorders^9^.

Protease/antiprotease imbalance, triggered by chronic lung inflammation, is advocated as a pathogenetic mechanism of emphysema in smokers^10^. This interpretation, however, fails to explain why only a fraction of smokers develop overt emphysema.

Reportedly, the risk of COPD is two to three times higher in smokers having a first-degree relative affected by the disease^11^. This suggests that genetic factors contribute to the development of COPD and related phenotypes^11^. As of now, deficiency of the serine protease inhibitor alpha-1-antitrypsin is the best known genetic factor linked to early-onset emphysema, but it occurs in 1 to 3% of the patients with COPD^12^.

In humans, lung size is directly proportional to body height^13^,^14^,^15^. With the present study, we examined the association between pulmonary emphysema and body height in a sample of 726 patients with spirometry-based diagnosis of COPD. Computed tomography (CT) was used as the reference diagnostic standard for emphysema. CT images were examined by three independent raters.

## Results

### Sample characteristics

The baseline characteristics of the study sample are given in Table 1. Seventy-three percent of the patients were males, 91% were either former or current smokers, and 81% had moderate to severe chronic aiflow obstruction.

Inter-rater reliability in scoring emphysema on CT was tested for by means of intraclass correlation coefficient (ICC). The estimated ICC was 0.9925 (95% confidence interval, 0.9916 to 0.9934), indicating excellent inter-rater reliability. Therefore, the scores of the three independent raters were averaged. Further details on inter-rater agreement are given in a supplementary file.

A diagnosis of emphysema was consistently made in 377 (52%) of 726 cases. Emphysema was rated mild in 66 (18%) of 377 cases, moderate in 168 (44%), and severe in 143 (38%). The frequency distribution of the severity scores of emphysema is shown in a supplementary file.

Patients with emphysema were significantly taller and thinner than those without (P < 0.001; Table 2). However, body mass index (BMI) did not vary significantly with body height. Emphysemic patients also featured significantly higher pack-years of smoking, and significantly worse aiflow obstruction than non-emphysemic ones (P < 0.001; Table 2). Similarly, the diffusing capacity of the lung for carbon monoxide (DL_CO_) was remarkably lower in patients with emphysema than in those without (P < 0.001; Table 2). There was an inverse linear correlation between DL_CO_ and the severity of emphysema on CT (r = −0.715; P < 0.001).

**Figure 1:**
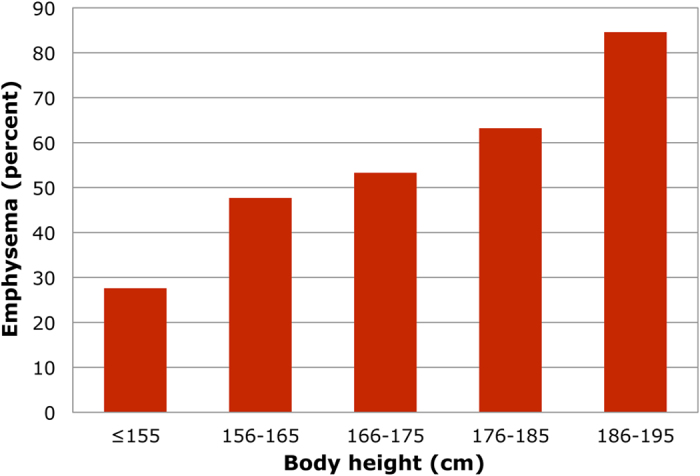
Prevalence of emphysema in 726 patients with COPD divided in five classes with 10-cm increase in body height. The prevalence of emphysema increases linearly across the classes (r^2^ = 0.96).

We divided the whole sample in five classes with 10-cm increase in body height, and measured the prevalence of emphysema in each of them. As shown in Fig. 1, the prevalence of emphysema rose linearly as a function of increasing body height.

With reference to gender-related differences, females in our sample were significantly shorter and thinner than males, and reported significantly fewer pack-years of smoking (Table 3). The degree of airflow obstruction was nearly identical between sexes, but the prevalence of emphysema was significantly less in females (P = 0.002). There was no statistically significant difference between males and females as regards the severity of emphysema on CT (Table 3).

### Predictors of pulmonary emphysema

Results of multivariate logistic regression are displayed in Table 4. The odds of emphysema was estimated to increase by about 5% (95% confidence intervals, 3 to 7%) along with one-centimeter increase in body height. Such an increase was highly statistically significant (P < 0.001), and remained nearly unchanged after adjusting for the potential confounders considered in the models.

In our sample, males were more frequent than females. So, we estimated the odds ratios for the risk of emphysema stratified by sex, applying the same logistic regression models as in Table 4. The odds ratios were comparable between sexes, after sampling error was factored in. The risk of emphysema was estimated to increase by about 5% (95% confidence intervals, 2 to 8%) per one-centimeter increment in body height in males, and by about 8% (95% confidence intervals, 4 to 14%) in females.

**Figure 2:**
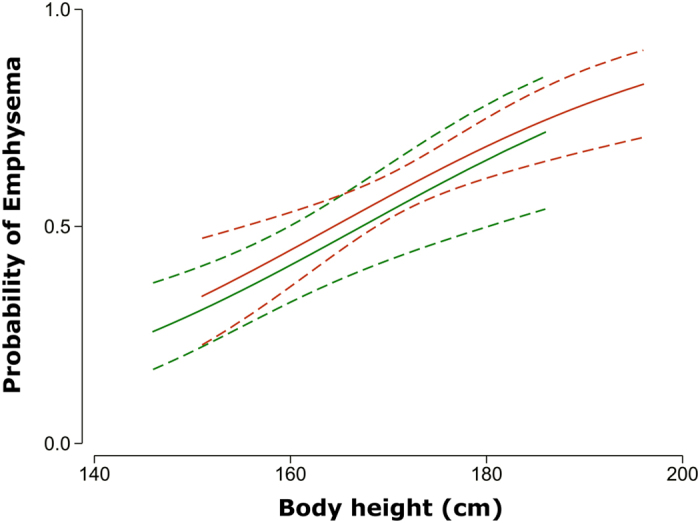
Predicted probability of emphysema across body height for males (red solid line) and females (green solid line) with age, body mass index, pack-years of smoking, and forced expiratory volume in one second set equal to the sample’s median values. Dashed lines indicate 95% confidence intervals.

The relationship between predicted probability of emphysema and body height is graphically displayed in Fig. 2, separately for males and females. The predicted probability was adjusted for age, BMI, pack-years of smoking, and forced expiratory volume in one second (FEV_1_) by setting the numerical values of these covariates equal to the sample’s medians.

In the subset of patients in whom DL_CO_ was measured (n = 527), the introduction of this covariate in multivariate logistic regression did not alter the odds for the risk of emphysema associated with increasing body height (odds ratio 1.05; 95% confidence interval, 1.02 to 1.09).

## Discussion

The relationship between body height and emphysema was first investigated by Thurlbeck and Haines^16^ in 1976. They examined the lungs of over 1,000 individuals at post-mortem, and found no correlation between body height and structural emphysema. In that study, however, the prevalence of COPD was unknown, and so was the cumulative smoking history. In addition, most of the lungs examined had only trace or mild emphysema. The results reported by Thurlbeck and Haines are plausible because their study is an “autopsy-population” survey^16^.

Should the likelihood of developing emphysema be dictated solely by increasing body height, in the absence of any inciting stimulus, then during the evolution of mankind there would have been a selective pressure favoring shorter individuals because emphysema carries substantial morbility, disability, and mortality.

We examined the relationship between body height and emphysema in white Caucasians with a firm diagnosis of COPD. In univariate analysis, the prevalence of emphysema rose linearly by 10-cm increase in body height. In multivariate analysis, the risk of emphysema increased by some 5% along with one-centimeter increase in body height, and remained virtually unchanged after adjusting for potential confounding variables. The odds for the risk of emphysema as a function of increasing body height were similar between males and females indicating that sex, in iteself, is not an independent predictor of emphysema.

We did, however, find that females had a degree of airflow obstruction nearly identical to that of males in spite of significantly lower pack-years of smoking. This finding is in agreement with previous reports, and lends support to the concept that females are likely more susceptible than males to the effect of cigarette smoking^17^,^18^,^19^.

A reduced BMI was significantly associated with the presence of emphysema. Such an association has been reported by others^4^,^5^,^6^,^7^, and is attributed to skeletal muscle atrophy and dysfunction due to disuse, malnutrition, comorbidities, and drugs (corticocosteroids)^20^. So, reduction of BMI appears to be a consequence of emphysema rather than a contributory cause.

In multivariate logistic regression, pack-years of smoking was not a statistically significant predictor of emphysema, suggesting that factors other than cigarette smoking are implicated in the pathogenesis of the disease. Data from two nation-wide surveys indicate that some 25% of the subjects with COPD in the United States are never-smokers^21^. Therefore, it would be desirable to assess the prevalence of emphysema, and its association with body height, in never-smokers with chronic airflow obstruction.

The relationship we observed between body height and emphysema in COPD is in all similar to that reported between body height and pulmonary infarction in the setting of acute lung embolism^22^. In that study, pulmonary infarction occurred in 31% of 335 patients with lung embolism diagnosed by CT angiograms. The prevalence of pulmonary infarction rose linearly with increasing body height, and the adjusted odds of infarction increased by some 5% per one-centimeter increase in body height^22^.

The finding of a nearly identical relationship between body height and two seemingly unrelated pulmonary disorders lends support to the hypothesis that the lungs of taller individuals are more susceptible to an inciting stimulus, be it blood-borne as in the case of infarction, or air-borne as in the case of emphysema. Notably, both infarction and emphysema manifest themselves in the peripheral regions of the lung.

Emphysema, at least at the very beginning, is predominantly distributed to the upper lobes^2^. It is well established that: (i) intrapleural pressure becomes less negative while moving from the top to the bottom of the lungs because of the compressing effect of the lung weight; (ii) transpulmonary pressure (the difference between alveolar and intrapleural pressure) increases from the bottom to the top of the lungs; (iii) accordingly, alveolar spaces in the apical regions are larger than those in the basal regions^23^.

Therefore, alveolar units in nondependent lung regions are more exposed to a mechanical stress, be it transient or persistent, than those at the lung base. Even more so if the lungs grow larger in size as it happens with increasing body height. In connection to this, it is worth recalling that individuals who develop primary spontaneous pneumothorax are usually tall and thin, and are frequently active smokers^24^. Focal subpleural emphysema is often detected by CT in the upper lung lobes^24^.

It is then conceivable that mechanical stress in the upper lung regions may be the initiating stimulus for the development of emphysema in the setting of chronic airflow obstruction. Mechanical stress may also compromise the alveolar microcirculation which could further impact on the development of emphysema, as originally postulated by Liebow^25^.

Finally, it should be mentioned that body height is a highly heritable polygenic trait^26^. Thus, costitutional factors, linked to the heritability of body stature, could make tall individuals more prone to develop emphysema or pulmonary infarction.

We acknowledge that our study has limitations. First, the sample size is relatively small, and originates from a single referral center. Second, our patients were all white Caucasians. Therefore, the observed association between body height and emphysema may not apply to individuals of other ethnic origin.

In sum, we found that body height is a strong, independent risk factor for emphysema in COPD. Further studies on the mechanical properties of the lungs in relation to increasing body height seem warranted.

## Methods

### Ethical approval

The study was carried out in accordance with the Code of Ethics of the World Medical Association (Declaration of Helsinki), and was approved by the institutional review board (Comitato Etico, Azienda Ospedaliero-Universitaria Pisana). Before entering the study, the subjects provided an informed written consent to let their clinical, spirometric, and radiologic data be used anonymously for the present analysis.

### Sample

The sample comprised 726 white Caucasian individuals of either sex, who were evaluated at the pulmonary outpatient clinic of the Institute of Clinical Physiology, and “Gabriele Monasterio” Tuscany Foundation, (Pisa, Italy) between November 1, 2001 and December 31, 2014. Criteria for recruitment into the study were: (a) firm clinical diagnosis of stable COPD; (b) airflow obstruction as indicated by a post-bronchodilator ratio of forced expiratory volume in one second over forced vital capacity (FEV_1_/FVC) <0.7; (c) post-bronchodilator change in FEV_1_ < 12% or <200 mL; (d) availability of chest computed tomography (CT) within two months of study entry. Patients were excluded if they had an established diagnosis of asthma, chronic lung disorders other than COPD, prior lobectomy or pneumonectomy, or active lung cancer. Patients were also excluded if they had had a clinically confirmed acute exacerbation in the 4 weeks preceeding the study entry.

### Study protocol

All the subjects were examined by board certified chest physicians. Clinical assessment included detailed clinical history, and physical examination. Smoking habits were recorded, and the cumulative cigarette consumption (pack-years) estimated. Body height and weight were measured, and body mass index (BMI) calculated accordingly. Lung function studies included the measurement of slow (SVC) and forced vital capacity (FVC), and of forced expiratory volume in one second (FEV_1_), before and after a metered-dose of inhaled bronchodilator (salbutamol, 400 μg). At least three spirometric measurements were obtained, and the highest values were chosen. The severity of COPD was ranked according to Global initiative for Obstructive Lung Disease (GOLD) guidelines as mild, moderate, severe, or very severe if FEV_1_ was >80%, 80 to 50%, 49 to 30%, or <30% of the predicted value, respectively^27^. The single-breath diffusing capacity of the lung for carbon monoxide (DL_CO_) was measured in 527 (73%) of 726 patients. Lung function studies were performed by experienced technicians according to ATS/ERS standards^28^.

### Lung Imaging

CT of the thorax was performed on multi-detector CT scanners with the patient breath-holding at full inspiration for 10 seconds. Acquisition setting was 120 kVp with mAs modulated according to the patient’s attenuation as assessed before scan acquisition (range, 60 to 250 mAs). Slice thickness was set at 0.65 mm. No contrast medium was infused. Scans were reconstructed in the axial, sagittal and coronal planes, and were imaged at a window level of –600 Hounsfield Units (HU) and width of 1,500 HU. Images had the identification data removed, were given a random code, and then examined by three raters (two radiologists and one chest physician) for the presence of areas of low attenuation and vascular disruption. The raters were blinded to clinical and lung function data. Maximum intensity projection technique was used to evaluate vascular disruption, and minimum intensity projection to highlight focal areas of low attenuation in the lung parenchyma^29^. The severity of emphysema was scored on a nonparametric scale from 0 (no emphysema) to 100 using the panel-grading (PG) method of Thurlbeck and coworkers^30^. This consists of 16 inflation-fixed, paper-mounted, midsagittal whole lung sections that are arranged at intervals of 5 between 0 and 50, and at intervals of 10 between 60 and 100. A score of 5 or less is consistent with trace emphysema, a score of 10 to 30 indicates mild emphysema, a score >30 to 50 moderate emphysema, and a score >50 to 100 severe emphysema. Such method was utilized in radiologic-pathologic correlation studies to validate the accuracy of CT in diagnosing emphysema *in vivo*^31^,^32^,^33^. In scoring emphysema on CT, the raters examined sagittal lung sections, and gave them the score of the standard most closely similar, or a score between two standards.

### Statistical Analysis

Patients’ baseline characteristics were compared across the groups (emphysema vs no emphysema, and males vs females) by Fisher’s exact test for the categorical variables. For the continuous variables, differences were tested for by Mood’s median test^34^.

The reliability of the three independent raters in scoring emphysema on CT was tested for by intraclass correlation coefficient (ICC)^35^. Inter-rater agreement was further evaluated by plotting the PG scores of each rater against the others in pairwise comparisons. The scatter plot was tested for departure from perfect agreement by fitting a simple linear regression model, and verifying the null hypothesis that the intercept is equal to zero and the slope is equal to one, jointly. Results are given in a supplementary file.

The association between the risk of emphysema and body height was estimated with logistic regression. We considered three regression models: a first model that included height as the only numeric predictor, a second model that additionally included age and sex, and a third model that additionally included BMI, pack-years of smoking, and FEV_1_ as percent predicted. We estimated the odds ratios and 95% confidence intervals associated with the predictors. We checked the linearity of the relationship between body height and the logit of the probability of emphysema by introducing splines variables. We did not find any evidence against linearity. A stratified analysis by sex was also performed. The statistical analyses were carried out using Stata release 13 (StataCorp LP, College Station, TX, USA).

## Additional Information

**How to cite this article**: Miniati, M. *et al*. Body height as risk factor for emphysema in COPD. *Sci. Rep.* **6**, 36896; doi: 10.1038/srep36896 (2016).

**Publisher's note**: Springer Nature remains neutral with regard to jurisdictional claims in published maps and institutional affiliations.

## Supplementary Material

Supplementary Information

## Figures and Tables

**Table 1:** Baseline characteristics of the study sample (n = 726).

Age, years	67	(61–72)
Male sex	533	(73)
Body height, cm	168	(162–174)
BMI, kg/m^2^	26.8	(23.5–29.9)
Never-smokers	67	(9)
Current smokers	244	(34)
Former smokers	415	(57)
Pack-years of smoking	38	(20–50)
GOLD stage		
I	66	(9)
II	371	(51)
III	214	(30)
IV	75	(10)
FEV_1%_ predicted	55	(41–68)
DL_CO_ % predicted*	66	(47–82)
Emphysema on CT	377	(52)
PG of emphysema	50	(35–60)

Data are medians (interquartile range) or numbers (percent).

BMI = body mass index; GOLD = Global initiative for Obstructive Lung Disease; FEV_1_ = forced expiratory volume in one second; DL_CO_ = diffusing capacity of the lung for carbon monoxide; CT = computed tomography; PG = panel-grading.

^*^In 527 (73%) of 726 patients.

**Table 2:** Baseline characteristics of patients with and without emphysema.

Characteristic	Emphysema				P-value
	Present (n = 377)		Absent (n = 349)		
Age, years	67	(61–72)	67	(61–72)	0.766
Male sex	295	(78)	238	(68)	0.002
Body height, cm	170	(164–175)	167	(160–172)	<0.001
BMI, kg/m^2^	24.8	(21.9–27.8)	28.7	(25.7–31.6)	<0.001
Pack-years of smoking	40	(29–53)	30	(15–46)	<0.001
FEV_1_, % predicted	45	(32–60)	64	(53–71)	<0.001
DL_CO_, % predicted*	53	(35–68)	80	(66–93)	<0.001

Data are medians (interquartile range) or numbers (percent). For abbreviations see Table 1.

^*^In 527 patients: 289 with emphysema, and 238 without.

**Table 3:** Baseline characteristics of the study sample split by sex.

Characteristic	Males (n = 533)		Females (n = 193)		P-value
Age, years	67	(62–72)	66	(58–71)	0.129
Body height, cm	170	(166–176)	160	(155–165)	<0.001
BMI, kg/m^2^	26.9	(23.9–29.7)	25.6	(22.2–30.1)	0.009
Pack-years of smoking	40	(29–55)	23	(7–40)	<0.001
FEV_1_, % predicted	55	(40–68)	55	(41–68)	0.933
Emphysema on CT	295	(55)	82	(42)	0.002
PG of emphysema	50	(35–60)	50	(35–65)	0.368

Data are medians (interquartile range) or numbers (percent).

For abbreviations see Table 1.

**Table 4:** Odds ratios for the risk of emphysema in logistic regression models.

	Model 1	Model 2	Model 3
Body height, cm	1.05 (1.03–1.07)*	1.05 (1.03–1.08)*	1.05 (1.03–1.08)*
Age, years		1.01 (0.99–1.03)^†^	1.02 (0.99–1.05)^†^
Male sex		0.93 (0.61–1.41)^†^	1.15 (0.69–1.93)^†^
BMI, kg/m^2^			0.82 (0.79–0-86)*
Pack-years			1.00 (0.99–1.02)^†^
FEV_1_, % predicted			0.96 (0.95–0.97)*

Values in brackets are 95% confidence intervals. For abbreviations see Table 1.

Model 1 includes body height as a single numerical predictor. Model 2 additionally includes age and sex. Model 3 additionally includes BMI, pack-years of smoking, and FEV_1_.

*P < 0.001; ^†^P > 0.05.
