# Supplementary material for: Body height as risk factor for emphysema in COPD
Source: Sci Rep. 2016 Nov 22;6:36896. doi: 10.1038/srep36896 (PMC5118794; doi:10.1038/srep36896)
Supplement: Supplementary Information [file srep36896-s1.pdf]

Body height as risk factor for emphysema in COPD

Massimo Miniati, MD, PhD, Matteo Bottai, ScD, PhD, Ivana Pavlickova, RN,

Simonetta Monti, MD, PhD.

Supplementary information

Emphysema grading on computed tomography

The severity of emphysema was assessed on computed tomography (CT) by the panel-grading method (PG) of Thurlbeck et al.[1] This consists of 16 inflation-fixed, paper-mounted, midsagittal whole lung sections that are arranged at intervals of 5 between 0 and 50, and at intervals of 10 between 60 and 100. A score of 5 or less is consistent with trace emphysema, a score of 10 to 30 indicates mild emphysema, a score >30 to 50 moderate emphysema, and a score >50 to 100 severe emphysema. In scoring emphysema on CT, the raters examined sagittal lung sections, and gave them the score of the standard most closely similar, or a score between two standards.

Inter-rater reliability in scoring emphysema on CT was tested for by means of intraclass correlation coefficient (ICC).[2] Results are given in the table below.

One-way analysis of variance for inter-rater agreement

Source of variation	SS	df	MS	F	Prob>F
Between raters	1717145.7	725	2368.4768	398.03	0.0000
Within raters	8640.1667	1452	5.950528		
Total	1725785.9	2177	792.73582		

SS = sum of squares; df = degrees of freedom; MS = mean squares.

$$ICC = Fobs–Fm / [Fobs + (g–1)Fm]$$

where Fobs is the observed value of F, Fm is the mean value of the F distribution, and g the number of raters. The estimated ICC was 0.9925 (95% confidence interval 0.9916 to 0.9934). The interpretation of the ICC is identical to the kappa statistic. Thus, the estimated ICC indicates excellent inter-rater reliability.

Inter-rater agreement was further evaluated by plotting the PG scores of each rater against the others in pairwise comparisons. The scatter plot was tested for departure from perfect agreement by fitting a simple linear regression model, and verifying the null hypothesis (H0) that the intercept is equal to zero and the slope is equal to one, jointly. Results are given in the table below.

Linear regression analysis of panel grading scores (pairwise comparisons)

Rater	Slope	95% CI	Intercept	95% CI
1 vs 2	1.003	0.994 to 1.012	0.144	-0.198 to 0.487
1 vs 3	0.995	0.986 to 1.004	0.168	-0.170 to 0.507
2 vs 3	1.001	0.992 to 1.010	0.163	-0.175 to 0.503

95% CI= 95% confidence interval.

The joint test P-values were as follows: 0.157 (rater 1 vs rater 2); 0.521 (rater 1 vs rater 3); 0.325 (rater 2 vs rater 3). Thus, the null hypothesis (H0) cannot be rejected at the level of alpha = 0.05.

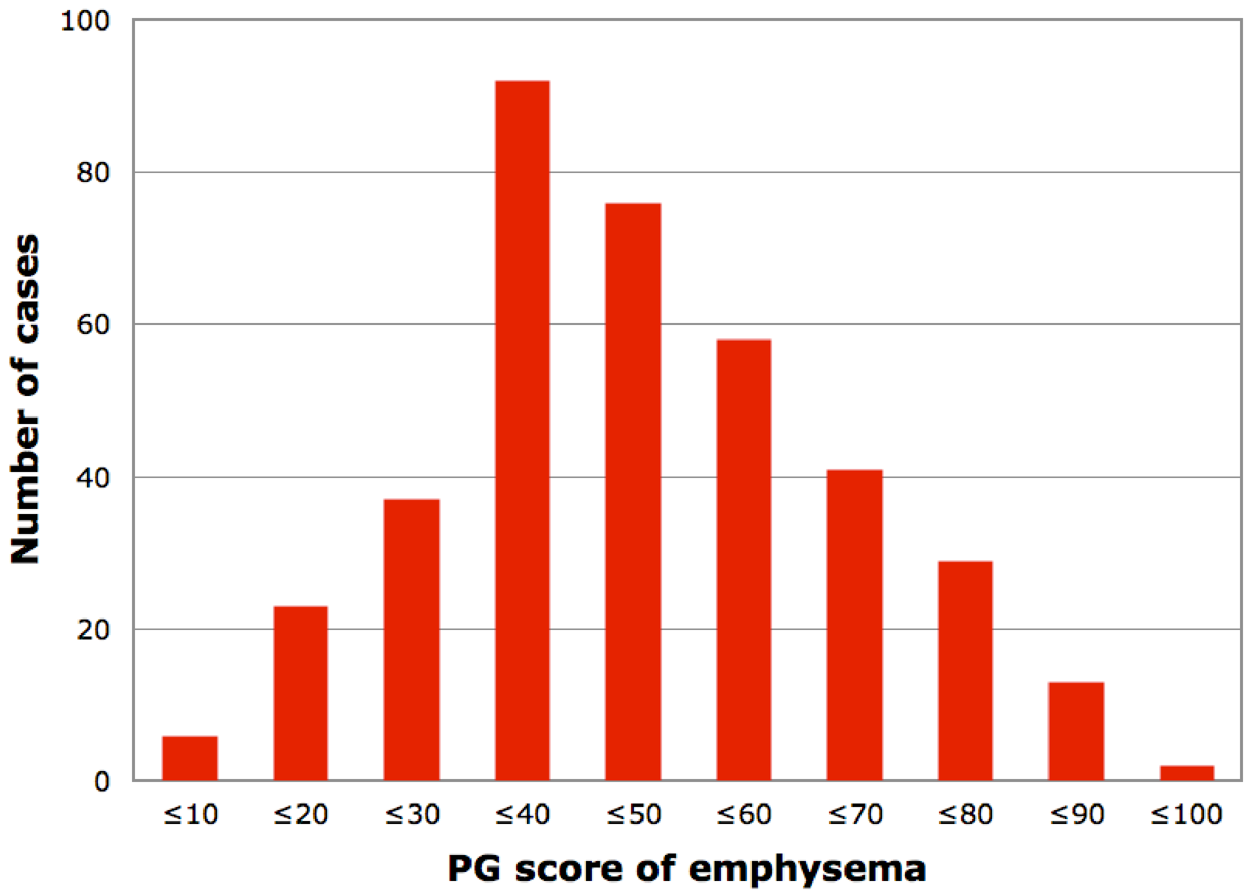

Figure 1. Frequency distribution of the PG scores in 377 patients with COPD who had evidence of emphysema on CT (PG>0).

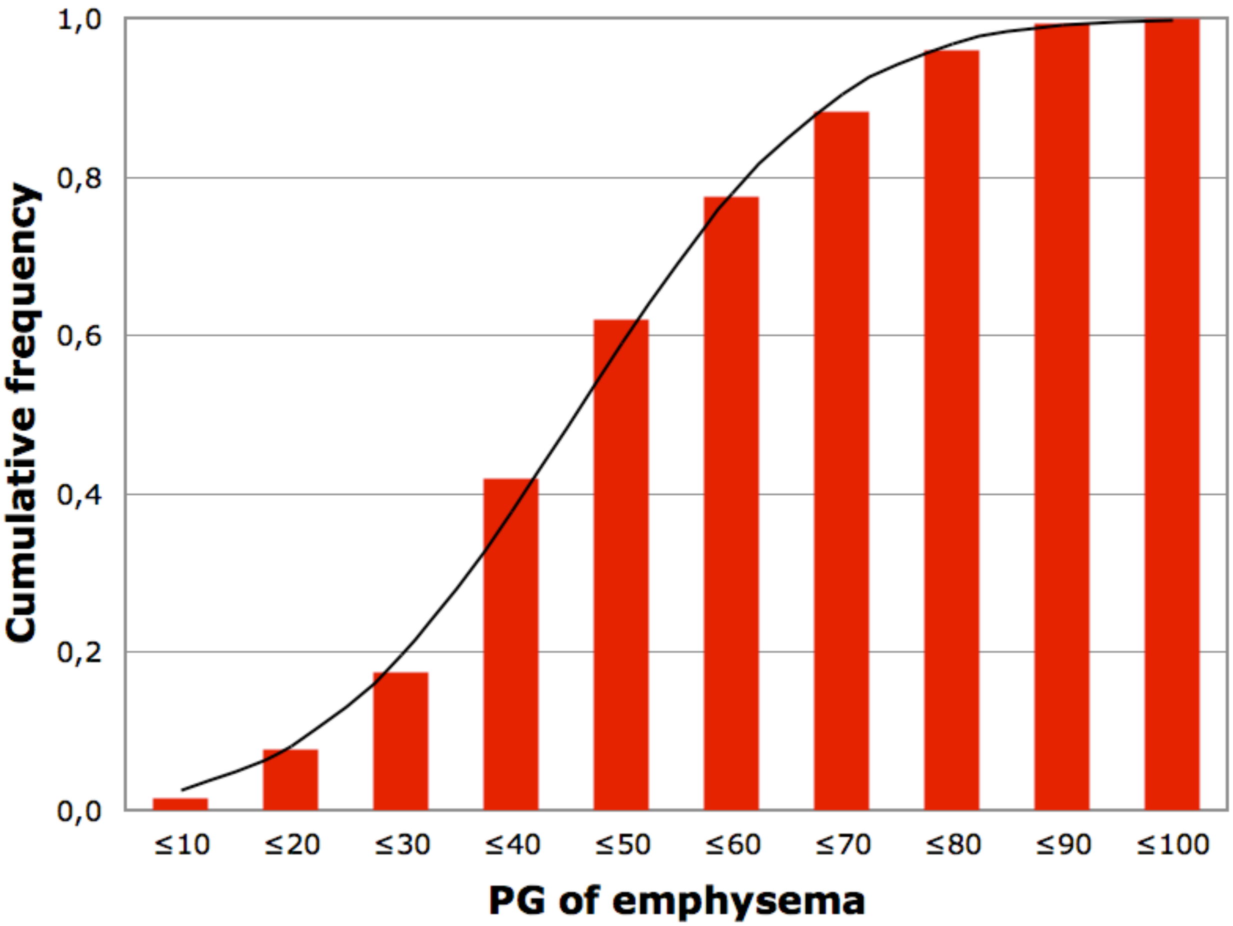

Figure 2. Cumulative frequency distribution of the PG scores of emphysema. The solid curve is the Normal cumulative frequency function. The observed frequency distribution of the PG scores is very similar to the Normal distribution. This indicates that our sample was representative of the whole spectrum of emphysema from mild to severe.

References

1. Thurlbeck, W.M., *et al.* A comparison of three methods of measuring emphysema. *Hum. Pathol.* **1**, 215-226 (1970).
2. Kish, L. *Survey sampling.* (New York: Wiley, 1965).
